# Green analytical method for determination of oxeladin citrate using advanced electrochemical modified sensors in pure form, pharmaceuticals, human serum and binary mixtures with guaifenesin

**DOI:** 10.1038/s41598-025-32960-5

**Published:** 2026-01-16

**Authors:** Sherin.F. Hammad, Hassan A. Hendawy, Hala. M. Habib

**Affiliations:** 1 Pharmaceutical Analytical Chemistry Department, Faculty of Pharmacy, Tanta University, Elgeish Street, Tanta, Egypt; 2 Egyptian Drug Authority (EDA), Giza, Egypt

**Keywords:** Oxeladin Citrate, Carbon paste electrode, Screen printed, Zeolite, Guaifenesin, Chemistry, Materials science, Nanoscience and technology

## Abstract

This work presents a selective, accurate, and highly sensitive differential pulse voltammetric (DPV) approach for the quantification of oxeladin citrate (OC). The electrochemical behavior of OC was investigated using several working electrodes, including carbon paste electrode (CPE), zeolite-modified carbon paste electrode (ZMCPE), iron oxide-modified carbon paste electrode (IOMCPE) and screen-printed multi-wall carbon nanotubes (SPMWCNTs), with operational parameters optimized through cyclic voltammetry. The results confirmed an irreversible, diffusion-controlled oxidation process for OC producing a peak between 0.7 and 0.8 V (vs. Ag/AgCl). The SPMWCNTs electrode outperformed the others, providing a linear calibration range from 0.35 to 3.9 μg/mL (R^2^ = 0.998), a detection limit (LOD) of 0.114 μg/mL, and a quantification limit (LOQ) of 0.35 μg/mL. The practical utility of the method was confirmed by the successful determination of OC in pharmaceutical products, both alone and in combination with Guaifenesin (GU), as well as in spiked human serum, all yielding excellent recovery values. The procedure’s environmental footprint was critically evaluated using the Analytical Greenness Calculator (AGREE) and the Complex Green Analytical Procedure Index (Complex GAPI), with the high scores obtained verifying its eco-friendly nature. Characterized by its sensitivity, operational simplicity, and sustainability. This voltammetric strategy offers a robust and viable alternative for the routine analysis of OC in pharmaceutical quality control and clinical settings.

## Introduction

**Fig. 1:**
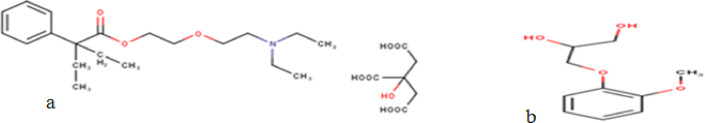
Molecular structure of OC (**a**) and GU(**b**).

Cough represents a highly common clinical symptom, leading to the widespread availability of numerous over-the-counter combination syrups for its treatment. A notable example is NEO-BRONCHOPHANE®, which is indicated for managing cough associated with acute and chronic bronchitis, as well as the common cold^1^. This formulation contains Oxeladin citrate an orally administered, centrally acting cough suppressant not related to opium or it’s derivatives so it is free of risk of dependence or addiction. Unlike opioid-based cough suppressants such as codeine, it does not exert its effects through opioid receptors . The primary mechanism through which Oxeladin Citrate works involves its action on the central nervous system, particularly targeting the cough center located in the medulla oblongata. The medulla oblongata is a part of the brainstem that plays a crucial role in the reflexive action of coughing. When irritants or foreign particles are detected in the respiratory tract, sensory nerves send signals to the medulla oblongata, which then triggers a reflexive cough to expel these particles. Oxeladin Citrate acts by modulating this reflex. It inhibits the nerve impulses that are responsible for initiating the cough reflex, thereby reducing the frequency and intensity of coughing. One of the key attributes of Oxeladin Citrate is its selectivity (Fig. 1a)^2^.

Although a few analytical techniques such as HPLC^3^, gas chromatography^4^, and potentiometry^5^ have been documented for OC, these approaches are often complex, laborious, and reliant on costly equipment. Furthermore, the literature reveals a complete absence of voltammetric sensors for OC quantification. Another component in NEO-BRONCHOPHANE® is Guaifenesin (GU, Fig. 1b), which functions as an expectorant^6^. Critically, no established procedure exists for the concurrent analysis of Oxeladin citrate and Guaifenesin in combined mixtures, pharmaceutical dosage forms or biological fluids. This gap underscores the demand for novel voltammetric techniques that are sensitive, selective, fast and straightforward to resolve this analytical need for determining both compounds in pure, pharmaceutical and biological samples. Voltammetry is an advantageous quantitative technique particularly because it permits the simultaneous measurement of multiple analytes within a single sample when their electrochemical signals are distinct^7^.

This work therefore focuses on creating new, simple and swift voltammetric procedures employing a range of contemporary modified electrodes and sensors: carbon paste electrode (CPE), zeolite-modified carbon paste electrode (ZMCPE), iron oxide-modified carbon paste electrode (IOMCPE) and screen-printed multiwall carbon nanotubes electrode (SPMWCNTs). The goal is to facilitate the direct measurement of Oxeladin citrate in its pure state, within pharmaceuticals, in serum and in binary mixtures with Guaifenesin. The CPE is widely used because its surface can be easily modified by adsorbing species from solution or forming oxide layers, it also provides a low background current, a wide potential window and high versatility^8^. Synthetic ZMCPE are increasingly prominent due to their unique chemical, physical and structural characteristics such as controllable shape and size, charge selectivity and significant ion-exchange capacity^9^. IOMCPE (magnetite, Fe₃O₄) integrate magnetic properties with nano-scale and surface phenomena, rendering them highly suitable for critical uses like chemical sensing and biological testing^10^.

The screen-printing technique is a highly promising method for the fast, simple and low-cost fabrication of biosensors, as it removes the requirement for electrode polishing between analyses. SPMWCNTs electrode in particular, demonstrate superior stability against electro-migration compared to other metallic materials, promote efficient electron transfer and are consequently extensively applied in the highly sensitive analysis of pharmaceutical substances^11^. In conclusion, innovating new voltammetric methods with diverse modified electrodes presents a viable strategy for the selective and fast quantification of Oxeladin citrate across various matrices. These methods are designed to surmount the drawbacks of current analytical techniques and allow for the combined assessment of Oxeladin citrate and Guaifenesin in both pharmaceutical products and biological fluids.

## Materials and methods

### Apparatus

A Metrohm 797 VA Electro-analyzers system equipped with Computrace software (version 1.3.1) was utilized to perform all voltammetric measurements. The setup featured a standard three-electrode cell, comprising a working electrode (of types specified later), an Ag/AgCl (3.0 M KCl) reference electrode and a platinum wire as the auxiliary electrode. A JENWAY 3510 pH-meter was used for all pH determinations. Characterization studies were performed using an AMETEK (EDAX) OCTANE PRO unit attached to a QUANTA FEG250 electronic microscope. Other instruments involved were a Sartorius analytical balance and an Eppendorf multipette plus micropipette. The entire experimentation was conducted under a controlled temperature of 25 ± 0.1 °C.

### Chemicals and reagents

All chemicals and reagents employed in this work were of analytical grade, and deionized water served as the solvent for all prepared solutions. Sigma-Aldrich supplied the reference standards for oxeladin citrate (OC, MW: 527.6 g/mol) and Guaifenesin (GU, MW: 198.22 g/mol). The commercial pharmaceutical formulation NEO-BRONCHOPHAN® (Batch No.: 1806293) was obtained from a local pharmacy. As per the product information, every 100 ml of this syrup contains 1.0 g of Guaifenesin, 200 mg of Oxeladin Citrate, and 100 mg of Diphenhydramine.

### Preparation of electrolyte solution

The supporting electrolyte, a 0.04 M Britton-Robinson (BR) buffer was formulated using glacial acetic acid (RANKEM, R190H21), orthophosphoric acid (SUPELCO, 100573), and boric acid (PIOCHEM, B20204245). The pH of this buffer solution was then modified to values between 5 and 10 by adding a 0.2 M sodium hydroxide solution (CDH, 300316).

### Preparation of standard stock

For every testing sequence a fresh 1 × 10⁻^3^ M standard stock solution of OC was formulated by dissolving 26.4 mg of its standard in 50 ml of deionized water. In a parallel manner, a 1 × 10⁻^3^ M GU stock solution was prepared by dissolving 9.9 mg of its standard in 50 ml of deionized water. These stock solutions were found to be stable for up to one week when kept under refrigeration.

### Preparation of working electrodes

#### Unmodified carbon paste electrode (CPE)

Fabrication of the unmodified carbon paste electrode followed an established protocol^12^. In summary, 0.5 g of graphite powder (20 µm, Sigma-Aldrich) was blended homogenously with 0.3 mL of paraffin oil using a mortar until a uniformly wetted paste was obtained.

#### Zeolite-modified carbon paste electrode (ZMCPE)

The zeolite-modified electrode was prepared by first dry-mixing 50 mg of zeolite powder with 0.45 g of graphite powder. This dry mixture was subsequently homogenized with about 0.3 mL of paraffin oil to yield a consistent paste.

#### Iron oxide-modified carbon paste electrode (IOMCPE)

Preparation of the iron oxide-modified electrode involved an initial dry-mixing step of 50 mg of iron oxide powder with 0.45 g of graphite powder^13^. Following this, 0.3 mL of paraffin oil was added, and the components were mixed thoroughly to form a homogeneous and wetted paste.

The paste from each of these electrode preparations was firmly compressed into the cavity of a 3.0 mm diameter insulin syringe body, which contained a copper wire to establish an electrical contact. To ensure a smooth surface, the electrode tip was polished on filter paper and then rinsed with double-distilled water and BR buffer prior to each measurement.

#### Screen-printed multiwall carbon nanotubes electrode (SPMWCNTs)

A commercial screen-printed electrode incorporating multiwall carbon nanotubes was sourced from Metrohm Middle East FZC (Batch: SPE-DEMO E0028/47) and employed without any further modification.

### Preparation of pharmaceutical dosage form and biological samples

To prepare the pharmaceutical sample for analysis, a 0.250 ml portion of NEO-BRONCHOPHAN® syrup was pipetted into a 50 ml volumetric flask and made up to volume with methanol^14^. The solution was subjected to 15 min of sonication to achieve full dissolution, after which it was filtered and the residue was rinsed with methanol. The final pharmaceutical stock solution contained OC at 10 μg/ml and GU at 50 μg/ml.

Human serum samples for the biological analysis were kindly donated by healthy volunteers. 1.5 ml Human serum was collected and mixed with 0.3 ml of 5% zinc sulfate solution and 0.5 ml ethanol and mixed by either shaking or vortexing to achieve proper homogenization and then subjected to centrifugation at 13,000 rpm to about half an hour to remove possible interference. After that, the supernatant was collected and diluted with 14 ml of B-R buffer at pH 8.0 prior to the voltammetric analysis. analytes free plasma samples were spiked with ascending known increments of standard stock solution then following the recommended procedures^15,16^.

### Experimental design

In the voltammetric procedure, measured volumes of the drug solution were added to a 15-ml graduated cylinder and the volume was completed with 0.04 M BR buffer. A pre-concentration step was then carried out by applying a 0.0 mV potential for 10 s to the solution, which was being stirred at 2000 rpm at room temperature. After stopping the stirrer, the system was allowed to equilibrate for 5 s before the voltammetric scan was started. To attain a reproducible and stable surface, every electrode was conditioned before use. This was done by running successive cyclic voltammetry scans from + 400 to -1400 mV at a scan rate of 100 mV/s in the pure buffer solution until a stable background signal was recorded.

## Results and discussion

### Morphological analysis of the fabricated electrodes

**Fig. 2:**
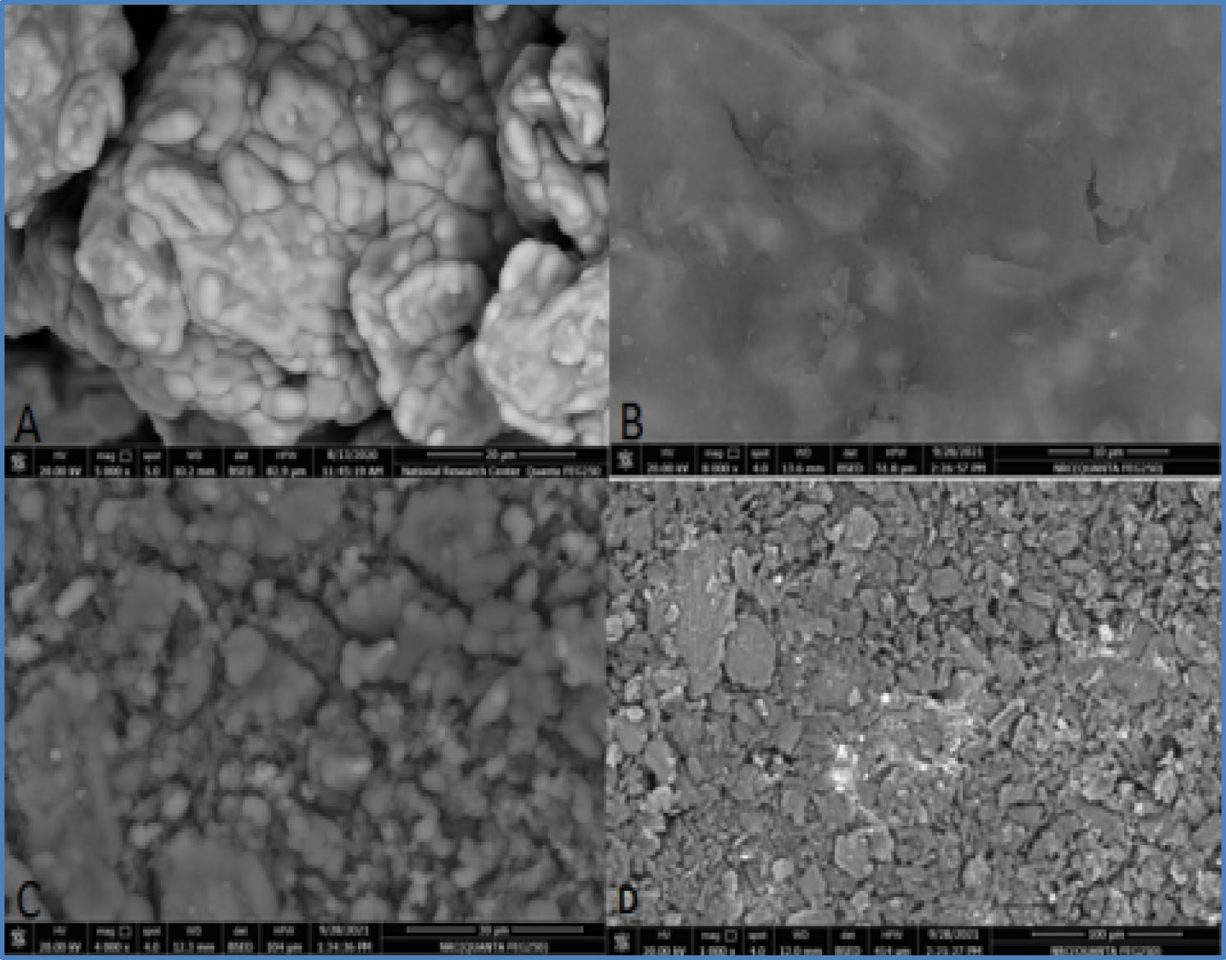
SEM images of CPE (**A**), SPMWCNTs electrode (**B**), ZMCPE (**C**) and IOMCPE (**D**).

**Fig. 3:**
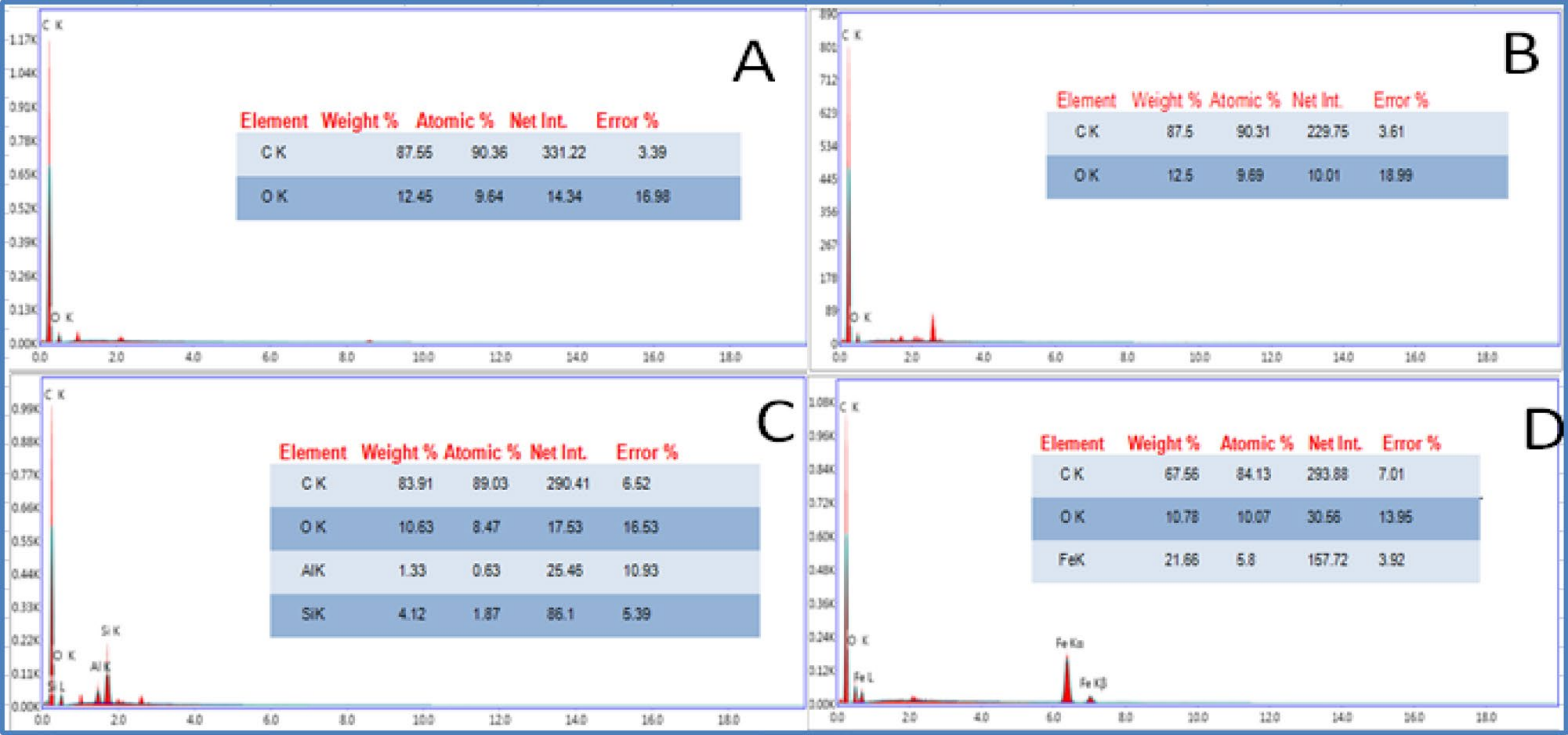
EDX spectrum of CPE (**A**), SPMWCNTs electrode (**B**), ZMCPE (**C**) and IOMCPE (**D**).

The morphological and elemental characteristics of the fabricated electrodes are displayed in the scanning electron microscopy (SEM) micrographs and energy dispersive X-ray (EDX) spectrometry data shown in (Figs. 2, 3)^17^. The performance of an electrochemical sensor is heavily influenced by its surface morphology. Examination of the unmodified CPE via SEM reveals graphite flakes that are irregularly shaped, with discontinuous grains and a poorly defined crystalline nature. In contrast, the SPMWCNTs electrode exhibits a rough, densely packed and highly porous structure. The images verify a uniform dispersion and strong adhesion of the carbon nanotubes to the underlying substrate indicating the presence of robust interfacial interactions that are vital for highly efficient electrochemical sensing^18^. Such a homogeneous spread of nanotubes over the electrode surface is a fundamental requirement for achieving dependable and reproducible performance from the modified electrode.

In the case of the ZMCPE the SEM micrographs indicate a uniform layer of zeolite nanoparticles deposited on the carbon paste surface where the particles themselves are nanoscale in size. The images also show that the zeolite nanoparticles adhere excellently to the electrode material, a feature that promotes effective sensor function. This consistent surface coverage ensures the modified carbon paste electrode behaves predictably^19^. Conversely, the SEM image for the IOMCPE indicates particles with irregular shapes and a propensity to agglomerate. EDX analysis was used to confirm the elemental composition, successfully identifying carbon, iron, and zeolite constituents in their corresponding electrodes. Together, the results from SEM and EDX provide vital insights into the physical structure and elemental makeup of the modified electrodes highlighting their significance in the development of capable electrochemical sensors. A critical consideration is that SEM images are often corrupted by electronic noise which can hinder accurate digital processing and interpretation. Consequently, noise reduction is an essential preprocessing measure^20^. In this study, denoising of the images was performed using Mathematica 8.0.1 software, applying the Laplacian model which is a standard representation for noise in SEM imagery. EDX spectroscopy served as a complementary technique to ascertain elemental composition, morphological traits and the dispersion of nanoparticles. The resulting EDX spectra showed clear peaks that were unique to the specific elements present in each electrode. A predominant carbon peak was observed for both the CPE and SPMWCNTs electrodes, verifying their carbon-based nature. The electrode modified with iron oxide nanoparticles (a graphite-iron oxide composite) displayed prominent peaks for carbon, oxygen, and iron, confirming the successful incorporation of these elements. Likewise, the EDX spectrum obtained for the ZMCPE contained distinct peaks for carbon, oxygen, aluminum and silicon, thereby validating its multifaceted composition.

### Electrochemical characterization of the electrodes

Cyclic voltammetry was employed to evaluate the electroactive surface area of every electrode, using a 1.0 mM K_4_Fe(CN)_6_ solution as a redox probe and measuring at various scan rates. The surface area was calculated by applying the Randles–Sevcik equation^21^:

$${\mathrm{I}}pa = ({2}.{69} \times {1}0^{{5}} ){\mathrm{n}}^{{{3}/{2}}} \times {\mathrm{A}} \times {\mathrm{C}}_{0} \times {\mathrm{D}}^{{{1}/{2}}} \times \upsilon^{{{1}/{2}}}$$

In this equation, I*pa* represents the anodic peak current (μA),n is the number of electrons transferred, A is the surface area of the electrode (cm^2^), C₀ is the concentration of K₄Fe(CN)₆ (mol/cm^3^), υ is the scan rate (V/s), and D is the diffusion coefficient (which is 7.6 × 10⁻⁶ cm^2^/s for K₄Fe(CN)₆).

Given that n = 1 for the 1.0 mM K₄Fe(CN)₆ solution, the electrode surface area was determined from the slope of the plot of Ipa against υ^1^/^2^^22^. The resulting calculated surface areas were 0.015 cm^2^ for CPE, 0.05 cm^2^ for ZMCPE, 0.044 cm^2^ for the IOMCPE, and 0.095 cm^2^ for the Screen-printed MWCNTs electrode. For a typical Carbon Paste Electrode (CPE), the electroactive area is always smaller than the geometric area (0.07 cm^2^ ) due to the porous, particulate nature of the carbon paste. The paste is composed of graphite powder and a binder (paraffin oil) Only the tips and outer surfaces of the carbon particles exposed at the electrode-solution interface contribute to electron transfer. The SPMWCNTs electrode possessed the greatest electroactive surface area, a property that is consistent with its stronger peak current response for the target analyte, OC. Typically, modifying an electrode surface with nanomaterials acts to augment its effective surface area, which in turn enhances the electrode’s capacity for adsorption.

### Performance of modified electrodes

**Fig. 4:**
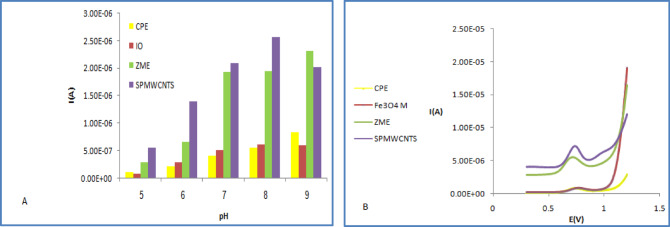
Comparison between different modified electrodes using DPV of OC in 0.04M B-R buffer in the pH range of 5–9 at scan rate 100 mVs − 1 (**A**), and pH 8 (**B**) which confirmed that SPMWCNTs electrode was the most sensitive with the highest current peak.

Comparative analysis of the anodic peak currents produced by the Carbon Paste Electrode (CPE), (ZMCPE), (IOMCPE) and Screen-Printed Multiwall Carbon Nanotubes (SPMWCNTs) electrode across a spectrum of pH values illustrated in (Fig. 4). The results demonstrate that the SPMWCNTs electrode produces a substantially higher anodic current than the other modified electrodes. This improved performance is a consequence of its increased electrochemical reactivity, a key trait that makes it particularly suitable for analytical purposes. As a result, the Screen-printed MWCNTs electrode was chosen to develop the validation methodology for the electrochemical assay of (OC). This includes its quantification in the pure state, in a pharmaceutical formulation, within spiked human serum, and when present in a binary mixture with Guaifenesin (GU).

### Influence of pH and supporting electrolyte

**Fig. 5:**
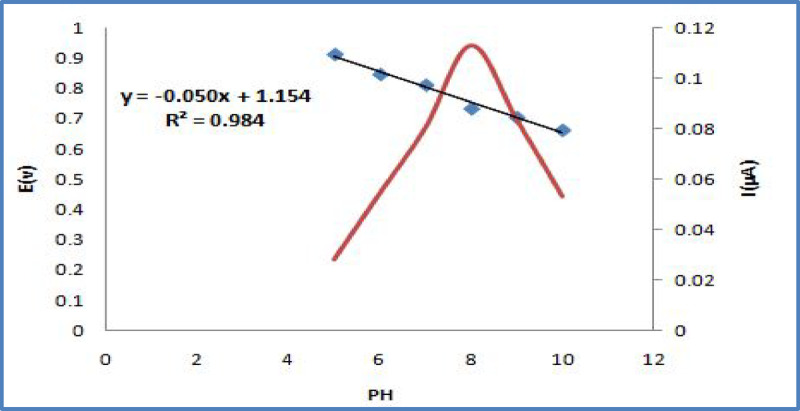
Effect of pH on peak current and peak potential for 1 × 10^−4^ M OC solution in 0.04 M B-R buffer at SPMWCNTs electrode.

**Fig. 6:**
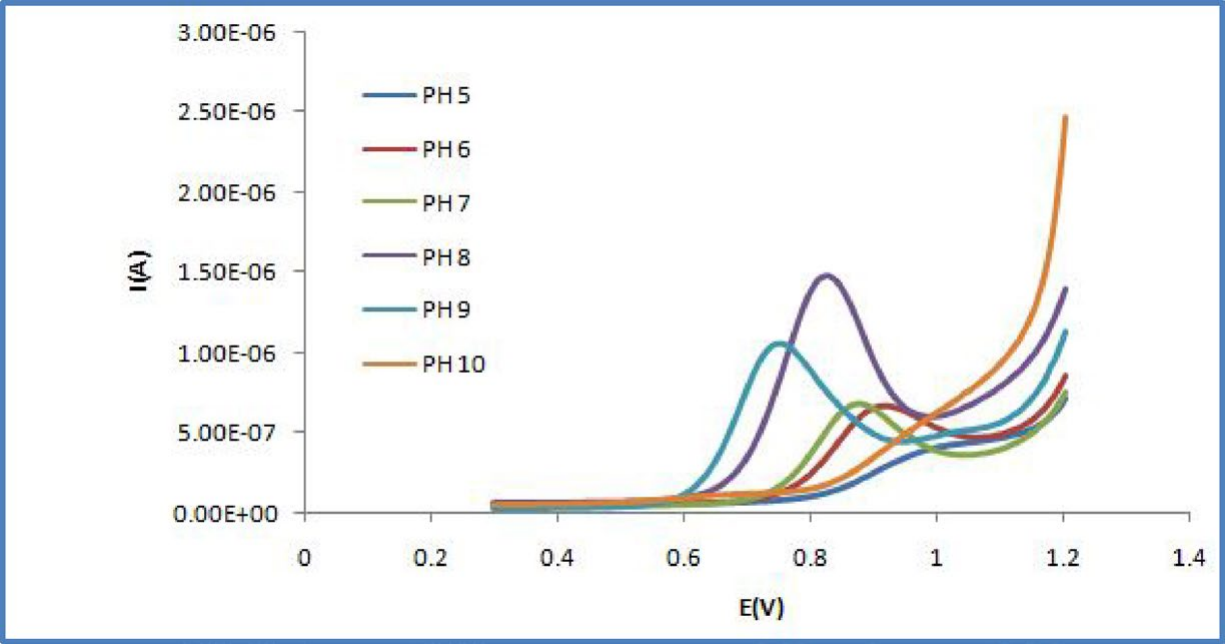
Differential pulse voltammograms for OC in 0.04 M B-R buffer at different pH at SPMWCNTs electrode.

The pH of the electrolyte solution is a fundamental factor with a substantial influence on the voltammetric signal. Therefore, the electrochemical activity of OC was examined over a pH spectrum from (5.0 to 10.0) as illustrated in (Fig. 5). Changes in the peak current and peak potential were monitored using a 1 × 10^−4^ M OC solution in a 0.04 M Britton-Robinson (B-R) buffer. The data revealed that the maximum peak intensity was achieved at pH 8.0. This pH was selected as the optimum for all further analyses because it yielded sharp and well-resolved peaks (Fig. 6).

The relationship between the solution’s pH and the peak potential was also studied. A consistent negative shift in the peak potential was noted with increasing pH, a trend that signifies the involvement of protons in the electrochemical process^23^. A linear regression analysis of peak potential versus pH provided a linear equation along with its correlation coefficient. The slope of this line was determined to be (-0.050 mV/pH) at the SPMWCNTs electrode. This measured slope aligns closely with the theoretical Nernstian value, indicating that the electron and proton transfer numbers in the electrode reaction are equal^24^. In conclusion, this investigation into pH dependence highlights the importance of precise pH regulation for achieving accurate and repeatable results, while also offering valuable information about the underlying reaction mechanism^25^.

### Scan rate analysis

**Fig. 7:**
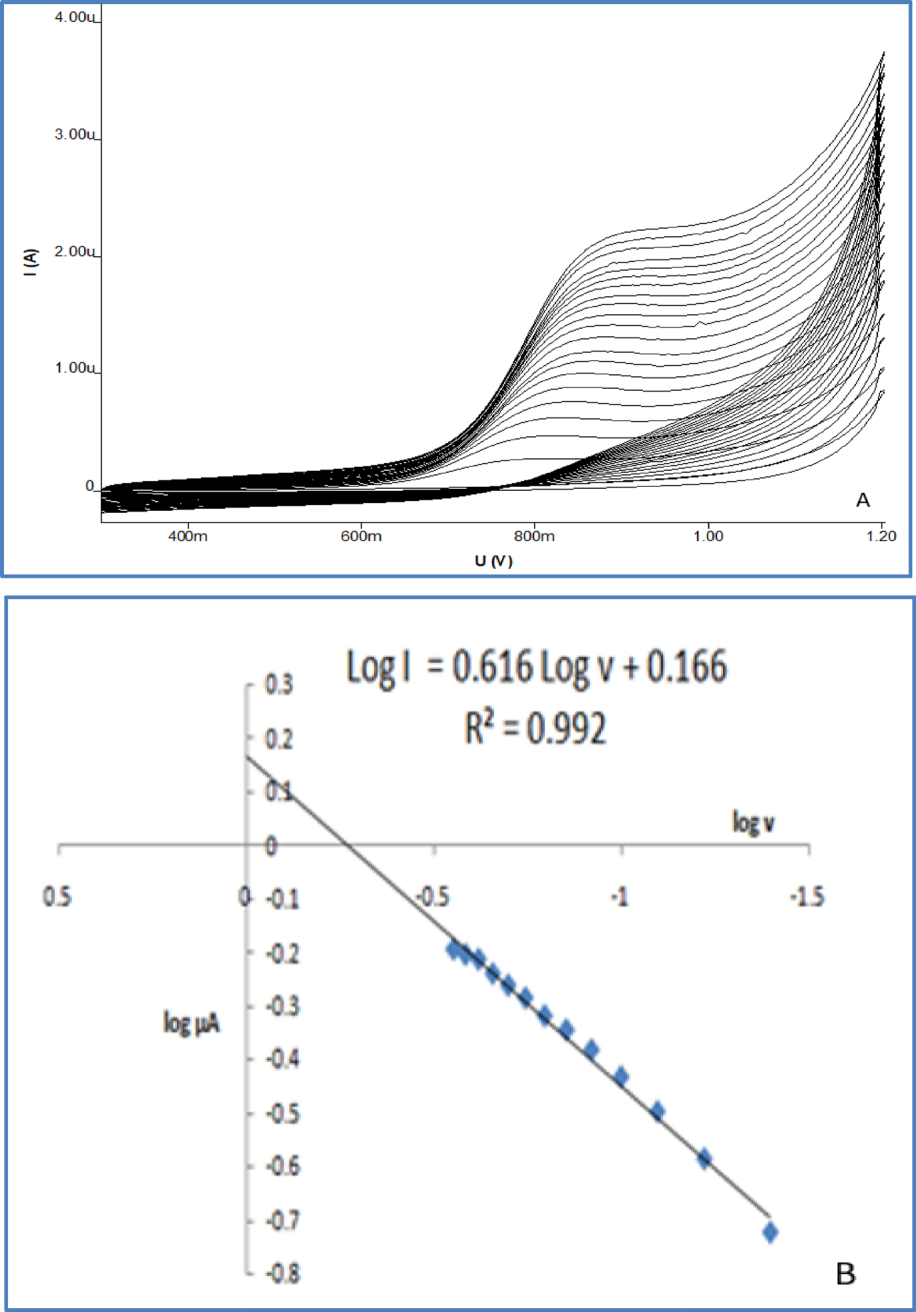
Voltammograms of OC response in 0.04 M B-R buffer at pH (8) at different scan ranging from 20 to 400 mV/s at SPMWCNTs electrode (**a**), The relationship between the logarithm of scan rate and the logarithm of the anodic peak current (**b**).

To understand the nature of the electrochemical process of OC at the SPMWCNTs electrode, the scan rate was varied to differentiate between a process controlled by adsorption and one controlled by diffusion. Such studies are essential for determining the reaction kinetics. (Fig. 7a) illustrates the effect of the scan rate (υ) on the peak current and peak potential. A progressive positive displacement of the anodic peak potentials concurrent with rising scan rate was detected, which is typical of an irreversible oxidation reaction for OC^26^.

As presented in (Fig. 7b), the logarithm of anodic peak current (Ipa) shows a linear relationship with the logarithm of the scan rate (υ). This dependence is expressed by the following mathematical equation:

$${\mathrm{Log}}\;{\mathrm{I}}\;\left( {\mu {\mathrm{A}}} \right) = 0.{616}\;{\mathrm{log}}\;\upsilon \;\left( {{\mathrm{vs}}^{{ - {1}}} } \right) + 0.{166}\;{\mathrm{R2}} = 0.{992}$$

The direct proportionality of the logarithm of anodic peak current (Ipa) to the logarithm of the scan rate provides strong evidence that the electrochemical oxidation is primarily a diffusion-controlled phenomenon. This finding indicates that the rate at which analyte molecules travel to the electrode surface is a major factor governing the electrochemical signal. Additionally, a linear plot with a slope of 0.616, this experimentally derived slope is near the theoretical value of 0.5 expected for a purely diffusion-controlled system, thus consolidating the finding that diffusion is the principal controlling mechanism at the SPMWCNTs electrode^27^.

### Suggested reaction mechanism

**Fig. 8:**
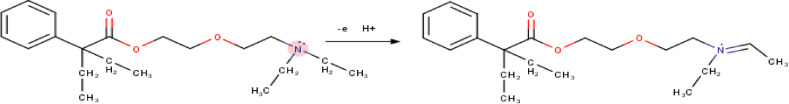
The suggested mechanism for oxidation of OC.

Considering the collective experimental findings, a reaction mechanism for the electrochemical oxidation of OC is suggested, which involves the transfer of one electron and one proton. This proposed mechanism, shown in (Fig. 8), suggests that the oxidation process consists of the concurrent release of one electron and one proton from the OC molecule, resulting in the generation of its oxidized form. The particular potential required for this transformation is specified by the operating potential used during the voltammetric analysis.

### Quantification of pure OC at SPMWCNTs electrode

**Fig. 9:**
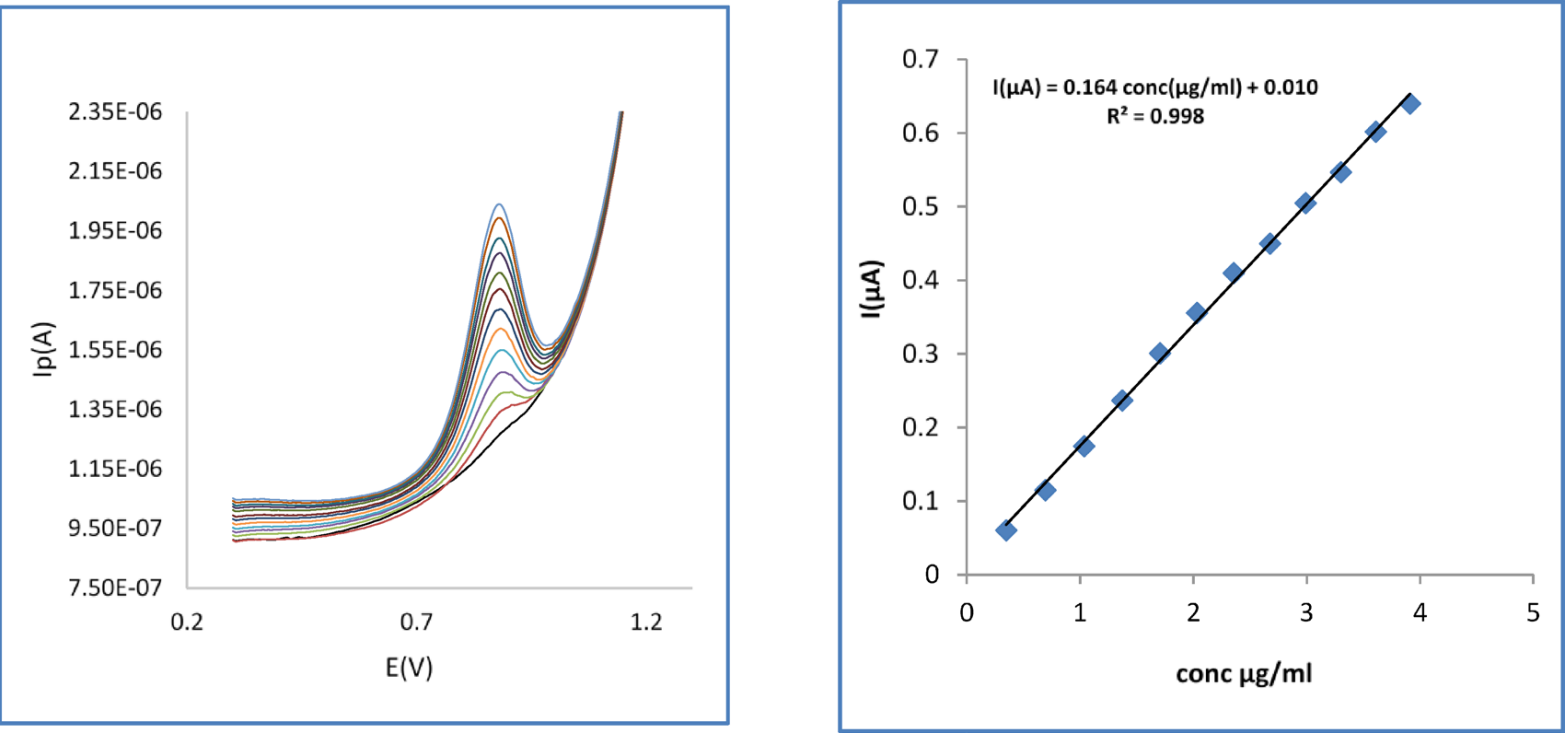
DPV and the corresponding calibration curves for successive additions of OC in 0.04 M B-R buffer pH (8) at pulse amplitude 50 mV, Pulse time(s) 0.04, Voltage step (v) 0.06, Voltage step time (s) 0.1 at SPMCNTs electrode.

A sensitive voltammetric approach for measuring OC was developed using the Differential Pulse Voltammetry (DPV) method. The measurements were carried out in a 0.04 M B-R buffer at the ideal pH of 8.0. Calibration graphs were generated by graphing the drug concentration (µg/ml) versus the measured anodic peak current I (µA), presented in (Fig. 9). The key statistical data for the calibration line are provided in Table 1.

**Table 1:** Statistical parameters for determination of OC using DPV at SPMCNTs electrode.

Parameters	Value*
Linear range conc (µg/ml)	0.35–3.9
Intercept	0.010
Slope	0.164
R^2^	0.998
RSD	1.16
LOD (µg/ml)	0.114
LOQ (µg/ml)	0.35

* Obtained from an average of six experiments.

### Determination of OC in pharmaceutical dosage form (syrup)

The practical utility of the developed voltammetric procedure was assessed by determining (OC) in a commercially available syrup (NEO-BRONCHOPHANE®). The assay was conducted with the SPMWCNTs electrode in a 0.04 M B-R buffer at pH 8.0. As presented in Table 2, the method produced outstanding recovery rates for samples fortified with specific OC amounts, verifying its effectiveness for use in pharmaceutical quality control.

**Table 2:** Recovery results of pharmaceutical dosage form of OC by the proposed DPV under optimum conditions compared to the Reported HPLC method.

	The proposedvoltammetric method	Reported HPLCmethod^3^
Added Conc (µg/mL) *	% Found	
0.69	99.3	99.0
1.04	100.5	100.6
1.37	100.9	100.1
Mean ± SD	100.26 ± 0.78	100.2 ± 1.06
t-Test	0.04	
t-Tabulated	2.78	
F-Test	1.62	
F-Tabulated	19	

*Each concentration value is average of three replicates.

### Application to spiked human serum

The applicability of the developed method for biological monitoring was evaluated by determining OC in human serum samples that had been spiked with the analyte. The outcomes of this investigation are summarized in Table 3. The accuracy of the procedure was evaluated via recovery tests, which yielded values very near 100%, attesting to the method’s high accuracy. Moreover, the consistency of the method was verified by carrying out triplicate analyses on separate OC solutions, which showed outstanding repeatability. These results collectively demonstrate the method’s reliability for application in complex biological fluids such as serum.

**Table 3:** Assay of OC in spiked human serum at SPMWCNT by the suggested method.

Added conc (µg/ml)	2.35
Recovery *(%)	99.57 ± 0.56

*Each concentration value is average of six replicates.

#### Analysis of OC and GU binary mixture

**Fig. 10:**
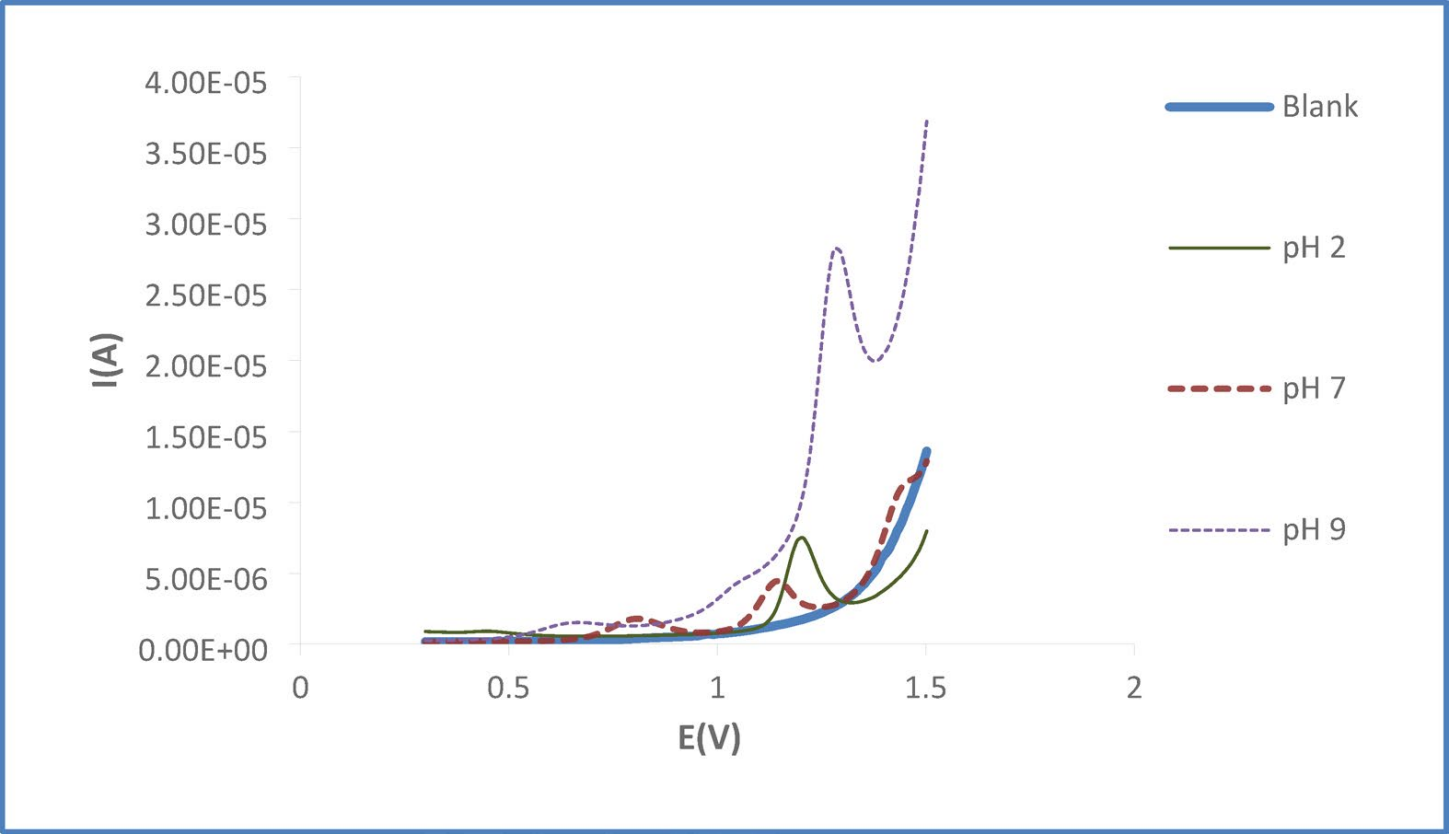
Differential pulse voltammograms of OC and GU mixtures in 0.04M B-R buffer pH (2,7,9) at SPMWCNTs electrode.

**Fig. 11:**
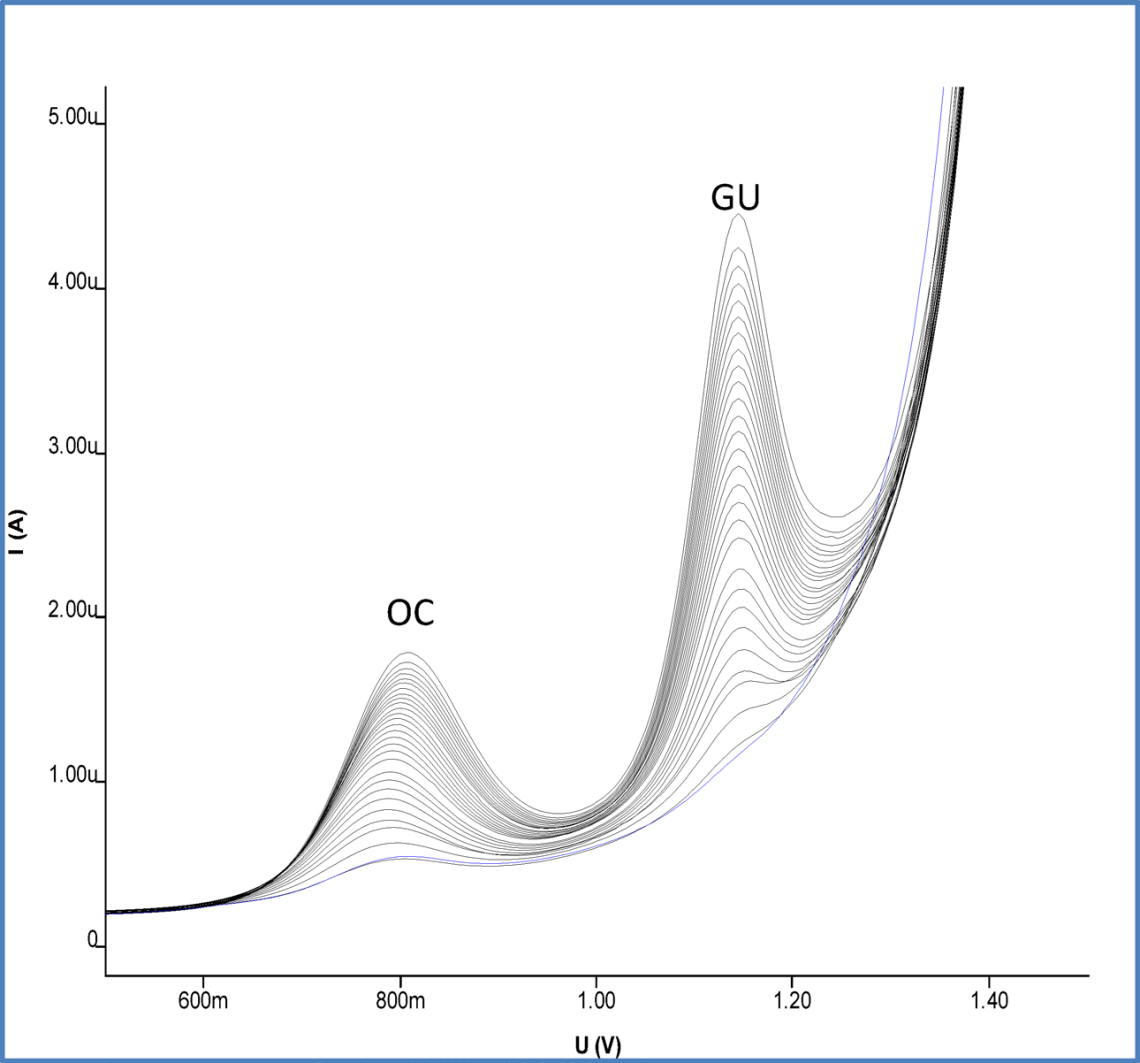
DPV voltammograms of OC and GU in their mixtures at pH 7 SPMWCNTs electrode.

In the NEO-BRONCHOPHANE® syrup, the level of Guaifenesin (GU) is considerably greater than that of OC. To facilitate the precise concurrent measurement of both compounds, the amount of OC in the test solutions was substantially raised. The effect of pH on the electrochemical signal of the binary mixture was explored over a pH interval from 2 to 9. From this evaluation, pH 7 was established as the most favorable pH for the simultaneous assay of the mixture in its pure state, within the syrup, and in biological specimens, as shown in.

(Figs. 10, 11). The statistical data for the successive standard additions of the mixtures in a 0.04 M BR buffer (pH 7) at the SPMWCNTs electrode employing DPV settings of a 0.050 V pulse amplitude, 0.04 s pulse time, 0.006 V voltage step, and 0.15 s voltage step time are listed in Table 4. The associated recovery percentages for the pharmaceutical formulation and the fortified serum samples are given in Tables 5 and 6.

**Table 4:** Statistical parameters for analysis of OC and GU binary mixture at pH (7) using DPV at SPMWCNTS.

Parameter	Values*	
	OC	GU
Linear range conc (µg/mL)	0.63–4	0.33–3.13
Intercept	0.2459	0.2337
Slope	0.1317	0.3249
R^2^	0.99	0.99
LOD(µg/ml)	0.27	0.06
LOQ(µg/ml)	0.63	0.20

* Obtained from an average of six experiments.

**Table 5:** Recovery results of pharmaceutical dosage form of simultaneous determination OC and GU by the proposed DPV at pH 7 at SPMWCNTs electrode.

	Added Conc (µg/mL) *	Recovery %
OC	1.7	99.41
	2.1	100.48
	2.5	100.80
GU	0.98	100.98
	1.30	99.33
	1.61	100.44

*Each concentration value is average of three replicates.

**Table 6:** Assay of OC and GU binary mixtures in spiked human serum at pH 7 at SPMWCNTs electrode by the suggested method.

	OC	GU
Added conc (µg/ml)	3.48	2.53
Recovery* (%)	99.71 ± 0.52	99.60 ± 0.54

*Each value is average of six replicates.

## Method validation

The newly developed analytical protocol was subjected to a thorough validation, evaluating the subsequent parameters as per the recommendations of the International Council for Harmonization (ICH)^29,30^.

### Linearity and range

A linear relationship was effectively confirmed between the anodic peak current and the concentration of OC, which is evidenced by the calibration graph in Fig. 9.

### Limit of detection (LOD) and limit of quantification (LOQ)

The LOD and LOQ for the analysis performed at the SPMWCNTs electrode were determined using the standard error of the calibration curve’s response and its slope^31^. These calculated values are displayed in Tables 1 and 4.

### Accuracy

To verify the method’s accuracy, the results were compared with those from a reference HPLC technique. A statistical comparison applying Student’s t-test and the variance ratio F-test indicated no noteworthy discrepancy between the outcomes of the two methods, as detailed in Table 2.

### Precision

The precision of the method was investigated through intra-day and inter-day assays. Newly prepared OC solutions with concentrations of 0.35, 0.69, and 2.03 µg/mL were analyzed three times within a single day and across three successive days. The mean recovery results of 99.70 ± 1.04, 99.86 ± 1.01, and 99.90 ± 1.00, achieved at the SPMWCNTs electrode, confirm the method’s high precision.

### Robustness

The method’s robustness was assessed by deliberately introducing small, controlled alterations to the analytical parameters. The impact of a modest pH shift (± 0.2 units) and a change in the measurement interval (10 ± 5 s) was studied. The percent recoveries stayed uniform at 99.68 ± 1.01 (at pH 8.2) and 99.59 ± 1.03 (at pH 7.8), proving the method’s resilience to slight, intentional changes in experimental conditions.

### Specificity

The procedure exhibited high specificity by precisely measuring OC in the presence of GU and other standard excipients found in the syrup. Significantly, no interference was observed from diphenhydramine, which is a third active component in the formulation. The method’s specificity received further support from its successful implementation in spiked human serum, where it attained high recovery rates with negligible interference from the matrix.

## Greenness estimation

There is a growing emphasis within the analytical chemistry field on creating the methods to be environmentally sustainable. This entails reducing the consumption of hazardous materials without compromising analytical quality. To systematically appraise environmental impact, a number of metric tools have been developed. These tools analyze aspects like reagent usage, chemical hazards, waste production, energy demand, ease of use, and automation potential^32^.

In this work, the environmental footprint of the newly devised method was appraised using the National Environmental Methods Index (NEMI), Eco-Scale Assessment (ESA), the Comprehensive Green Analytical Procedure Index (GAPI), and the Analytical Greenness Calculator (AGREE). The method attained an impressive AGREE score of 0.85 is much closer to the ideal score of 1.0 indicating a lower environmental and health impact overall.

This finding was supported by the GAPI evaluation, which produced a three-green symbol, affirming the technique’s minimal ecological impact. A summary of these results is provided in Tables 7, 8, 9.

**Table 7:** GAPI and AGREE tools for assessment of greenness values.

Applied instrument	GAPI	AGREE
Reported HPLC method^3^		
Proposed voltammetric method

**Table 8:** Assessment of the greenness of the developed voltammetric method and HPLC method using AGREE calculator.

Parameters	Developed voltametric method	Reported HPLC^3^
1- Select the sampling procedure	off-line	off-line
Enter the amount of sample in gm. or Ml	0.015 g	0.05g
What’s the positioning of the analytical device	Off-line	Off-line
How many major distinct steps are there in the sample preparation procedure? (sonication, mineralization, centrifugation, derivatization, etc.)	3 or fewer	3 or fewer
Degree of automation, sample preparation	Semi-automatic miniaturized	Semi-automatic, Not miniaturized
Select derivatization agent (if used)	Not used	Not used
Enter the amount of waste in g or mL	< 0.1g	< 0.1g
Number of analytes determined in a single run-sample throughput (sample analyzed per hour)	1 (analytes) 60	(analytes) 4
Select the most energy-intensive technique used in the method Total power consumption of a single analysis in kWh	< 0.001 kWh	0.1–1.5 kWh
Select the type of reagent	No reagent	All reagents are bio-based
Does the method involve the use of toxic reagents of solvents	No	Yes Acetonitrile–trifluoracetic acid
Select the threats which are not avoided	No threats	Highly flammable

**Table 9:** Assessment of the greenness of the developed voltammetric method and HPLC method using GAPI tool.

Parameters	Developed voltametric method	Reported HPLC^3^
5-Type of method /analysis	Simple procedure	Simple procedure
Amount	< 10 mL	< 10 mL
Health hazard	NFPA score = 0	Moderate toxic NFPA score = 2
Safety hazard	NFPA flammability is 0	Highest NFPA flammability or instability score of 2 or 3, or a special hazard is used
Energy	≤ 0.1 kWh per sample	≤ 1.5 kWh per sample
Occupational hazard	Hermetic sealing of analytical process	Hermetic sealing of analytical process
Waste	1–10 mL	> 10 mL
Waste treatment—type of analysis	No treatment Qualitative and quantitative	No treatment Qualitative and quantitative

In conclusion, the findings from the green metric assessments definitively indicate that the proposed voltammetric approach is more ecologically sound than traditional methods. This makes it an appropriate and conscientious option for the regular quality control and analysis of the target pharmaceutical compound.

## Conclusion

In conclusion, this research has effectively designed a sustainable and high-performance electrochemical sensor based on a screen-printed multi-walled carbon nanotube (SPMWCNTs) platform for the accurate measurement of oxeladin citrate (OC). The sensor exhibited outstanding performance in diverse matrices, such as the pure active compound, commercial dosage forms, human serum and a binary mix with Guaifenesin (GU). The electrochemical reaction was characterized as an irreversible, diffusion-controlled oxidation, generating a well-defined and stable anodic peak. The SPMWCNTs electrode offered distinct benefits compared to the other materials tested, attributable to its greater electroactive surface area, improved sensitivity and superior electrochemical signal. Its disposable, ready-to-use design also streamlines the analytical process and prevents carryover contamination though the recurring cost of single-use electrodes is a factor for budget considerations. The associated DPV methodology was validated and proven to be accurate, precise, and highly sensitive, allowing for the dependable assay of OC without any interference from typical formulation additives. When this analytical performance is coupled with a favorable green metric evaluation, the developed SPMWCNTs-based method stands out as an ideal, economical, and environmentally sound solution for routine quality control applications, presenting a strong substitute for more intricate and costly conventional methods like HPLC.

## Data Availability

Most data generated or analyzed during this study are included in this published.
